# Notes and News

**Published:** 1899-04-29

**Authors:** 


### Notes and News.

(Collected and Communicated.)

### HOSPITAL MEETINGS.

**King's College Hospital.—A Festival Dinner will take place at half-past seven p.m., on Wednesday, May 3rd, at the Hotel Cecil. The Bishop of London in the chair.**

**Hospital for Sick Children, Great Ormond Street.—A Festival Dinner will take place at half past seven p.m., on Wednesday, May 3rd, at the Hotel Cecil. Viscount Peel in the chair**

A new ward has been added to the Sydney Western Suburbs Cottage Hospital. In our colonies the cottage hospital is found to be as successful and as popular as in England.

A complimentary dinner, at which about 70 guests attended, was given at Gosport to Mr. Thomas Naish Blake, in recognition of his munificient gift of an isolation hospital to the town.

The annual meeting of the Wolverhampton and District Hospital for Women will be held on Friday, April 28th, at half-past three, under the presidency of the Mayor.

The will of Baroness de Hirsch is the subject of much conjecture. The contents have not yet been officially announced, but it is believed that about 80 per cent. of her vast fortune has been bequeathed to charity, principally amongst the Jews in foreign cities.

A hospital ship for Colombo Harbour has been launched, built by order of the Colonial Government of Ceylon. It is built of teak, is 130 ft. by 25 ft., and resembles the East Indian trading vessels. The patients will be lodged between decks in a large ward.

Mr. John Robinson, J.P., has offered to build a new wing at the Nottingham Hospital, costing £10,000, in memory of his only son, Mr. J. Sandford Robinson. The generous gift, one of many others previously made, has been gratefully accepted.

It is hoped that the proposed convalescent home for the London Hospital will benefit to the extent of £400, from the proceeds of the brilliant and successful ball held through the initiation of Mrs. Spender, who has once more shown her ability to organise and carry into effect entertainments for the benefit of charity. The London Hospital is fortunate in having enlisted her sympathy, and doubtless is duly appreciative.

Dr. F. H. Waddy, on leaving the Fir Vale Infirmary, where he had been assistant resident medical officer, was presented with a case of surgical instruments from the nursing staff as a token of their esteem.

Messrs. Hughes (Limited) write to say that they have made arrangements which enable them to guarantee that every cow from which milk is drawn supplying their dairy is free from tuberculosis and is in good health.

In aid of the British Ophthalmic Hospital at Jerusalem, a café concert is to take place at the Grafton Galleries on Monday, May 8th, at which it is hoped H.R.H. the Prince of Wales, who is the Grand Prior of the Order of the Hospital of St. John of Jerusalem, will be present.

Dr. John Sibbald, Commissioner in Lunacy for Scotland, is to be presented with his portrait by his many friends and admirers in recognition of his long and notable services as Commissioner, and of the great work he has effected in the interest of the insane in Scotland. The portrait is to be painted by the President of the Royal Scottish Academy.

Lord Lister opened a ward at the Royal Southern Hospital, which is to be devoted to the work of the new school of tropical diseases at Liverpool. On the occasion the President of the hospital remarked that it had never been free from cases of tropical disease since opened, and one ward at the time was quite full of such cases. Lord Lister and the many distinguished speakers present at the ceremony were unanimous in expressing their conviction that a school for the study of tropical diseases was a most important and desirable institution.

There are many people quite unaware of the useful work accomplished outside the institution walls by many of our hospitals by means of a Samaritan Fund. In some cases the fund is a very small one, and the difficulty of selecting the most necessitous patients for assistance where the needs are so many is very great. Every hospital could most usefully continue its good work by caring for the convalescents if means were at hand. The breadwinner would be sooner fit for work if he had an opportunity of regaining his health in good air and comfortable surroundings, whilst the provision of warm garments would prevent the speedy return of many a patient to the hospital wards. St. Thomas's Hospital is fortunate in possessing an important Samaritan Fund, the distribution of which illustrates how really necessary such an adjunct is to the work of a hospital. Having a handsome income at their command, the authorities are able to supplement the medical treatment received in the hospital by providing in most cases what is necessary through change of air or suitable attire. It was found that upwards of 800 patients required help, and although the income of the fund exceeded £1,000 it was not sufficient for such large requirements. The result is that during the past year £24 more than the sum in hand was expended. So useful a part of hospital work must appeal to all. Hospitals are always ready to undertake the task of helping their patients when recovering from sickness if funds are entrusted to them.

